# Long‐Life Lithium‐Ion Sulfur Pouch Battery Enabled by Regulating Solvent Molecules and Using Lithiated Graphite Anode

**DOI:** 10.1002/advs.202302966

**Published:** 2023-09-15

**Authors:** Dan Huang, Zhicheng Wang, Ran Han, Shoulei Hu, Jiangyan Xue, Yumeng Wei, Haiqi Song, Yang Liu, Jingjing Xu, Jun Ge, Xiaodong Wu

**Affiliations:** ^1^ School of Nano‐Tech and Nano‐Bionics University of Science and Technology of China Hefei 230026 China; ^2^ i‐lab Suzhou Institute of Nano‐Tech and Nano‐Bionics (SINANO) Chinese Academy of Sciences Suzhou 215123 China

**Keywords:** ether electrolytes, graphite, lithium‐ion sulfur batteries, long cycle life

## Abstract

The development of lithium‐sulfur (Li‐S) batteries is severely limited by the shuttle effect and instability of Li‐metal anode. Constructing Li‐ion S batteries (LISBs), by using more stable commercial graphite (Gr) anode instead of Li‐metal, is an effective way to realize long‐cycle‐life Li‐S batteries. However, Gr electrode is usually incompatible with the ether‐based electrolytes commonly used for Li‐S batteries due to the Li^+^‐ether complex co‐intercalation into Gr interlayers. Herein, a solvent molecule structure regulation strategy is provided to weaken the Li^+^‐solvent binding by increasing steric hindrance and electronegativity, to accelerate Li^+^ de‐solvation process and prevent Li^+^‐ether complex co‐intercalation into Gr anode. Meanwhile, the weakly solvating power of solvent can suppress the shuttle effect of lithium polysulfides and makes more anions participate in Li^+^ solvation structure to generate a stable anion‐derived solid electrolyte interface on Gr surface. Therefore, a LISB coin‐cell consisting of lithiated graphite anode and S@C cathode displays a stable capacity of ≈770 mAh g^−1^ within 200 cycles. Furthermore, an unprecedented practical LISB pouch‐cell with a high Gr loading (≈10.5 mg cm^−2^) also delivers a high initial capacity of 802.3 mAh g^−1^ and releases a stable capacity of 499.1 mAh g^−1^ with a high Coulombic efficiency (≈95.9%) after 120 cycles.

## Graphical abstract

TFEE‐based electrolyte is fabricated by regulating molecular structure of DME solvent, which can weaken the Li^+^‐solvent binding energy and accelerate Li^+^ de‐solvation process, inherently preventing Li^+^‐ether complex co‐intercalation into Gr anode and inhibiting dissolution of lithium polysulfides (LiPSs). Hence, this electrolyte shows a good application potential in long‐life lithium‐ion sulfur batteries (LISBs) with S@C cathode and lithiated graphite (LG) anode.

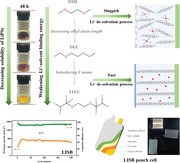

## 1 Introduction

The rechargeable lithium‐sulfur (Li‐S) batteries have aroused great concerns in recent years because S cathode not only theoretically delivers a high capacity of 1675 mAh g^−1^ and a high energy density of 2600 W h kg^−1^ upon complete reduction by lithium to form Li_2_S, but also is naturally abundant, cheap and environmentally friendly.^[^ ^1^, ^2^ ^]^ However, the development of Li‐S batteries still suffers from many issues. The most prominent issue is the use of a lithium metal anode. That inevitably gives rise to the growth of lithium dendrites, huge volume transformation, and serious interfacial side reactions, thus generating safety concerns and fast capacity decay because of a larger reactive surface area exposed to lithium polysulfides (LiPSs).^[^ ^3^ ^]^ In spite of many efforts have been made to solve the issues of lithium metal anode like using 3D structure current collectors to uniformize Li deposition behavior and relieve volume transformation,^[^ ^4^, ^5^ ^]^ constructing an artificial solid electrolyte interphase (SEI)^[^ ^6^, ^7^, ^8^ ^]^ or adding SEI‐forming additives in electrolytes^[^ ^9^, ^10^ ^]^ to inhibit interfacial side‐reactions between LiPSs and active lithium, the cycling lifespan of Li‐S batteries is still unsatisfactory.

Using Li‐free alternative anode to replace Li metal anode, such as carbon, Si, or Sn anode, is a very promising strategy to circumvent above problems.^[^ ^11^, ^12^, ^13^, ^14^ ^]^ However, high capacity Si or Sn anode still undergoes a large volume change, thus the corresponding Li‐ion S batteries (LISBs) comprising S cathodes and Si (or Sn) anodes still show low reversible capacity ascribed from an unstable interface like lithium metal.^[^ ^15^ ^]^ Hard carbon anode also faces many challenges in the long run, in terms of cycling reversibility, Coulombic efficiency (CE), and interfacial side reactions.^[^ ^16^ ^]^ Commercial graphite (Gr) anode is a kind of very successful anode for long cycle‐life Li‐ion batteries in ethylene carbonate (EC)‐containing electrolytes, because the very small volume change upon Li^+^ intercalation/deintercalation and superior SEI layer formed in the initial cycle.^[^ ^17^ ^]^ Unfortunately, carbonate electrolytes are not suitable for Li‐S battery because of the severe side reactions between LiPSs and carbonate solvents.^[^ ^18^ ^]^ The commonly used electrolytes for Li‐S batteries, such as 1 m lithium bis(trifluoromethanesulphonyl)imide (LiTFSI)−1, 3‐dioxolane (DOL)/1, 2‐dimethoxyethane (DME) (named DME‐based electrolyte), cannot tolerate Gr anode because the strong Li^+^‐DME binding energy brings sluggish Li^+^ de‐solvation process, thus DME solvents will be continuously co‐intercalated with Li^+^ into Gr interlayer to exfoliate the Gr lattice structure.^[^ ^19^ ^]^

Exploiting novel electrolytes with good compatibility with LiPSs and Gr anode is very significant for developing long‐life Gr anode‐based LISBs. Unfortunately, until now there are only few studies on Gr anode‐based LISBs, which nearly all focused on using high concentration electrolytes (HCE) or located high concentration electrolytes (LHCE) to inhibit the co‐intercalation.^[^ ^19^, ^20^, ^21^, ^22^ ^]^ However, this kind of electrolytes suffer from various drawbacks such as high cost, high density, low ionic conductivity, and environment unfriendly arising from large amount of Li salt used. Recently, the weakly solvating electrolytes have emerged as a promising candidate for lithium‐metal batteries (LMBs) and lithium‐ion batteries (LIBs), because the weakened solvating power of solvent can contribute to an anion‐derived interfacial chemistry, can effectively suppress lithium dendrite growth and prevent Li^+^‐solvent from inserting into Gr interlayers. ^[^ ^23^, ^24^, ^25^, ^26^ ^]^ For example, Yao et al. ^[^ ^25^ ^]^ proposed a weakly solvating electrolyte by using a cyclic ether solvent (1,4‐dioxane), the weakly solvating ability of solvent with Li^+^ promotes the involvement of more anions in the Li^+^ solvation structure, thus induces a helpful anion‐derived SEI layer and achieves a stable cycling of Gr electrodes. Inspired by these works, by reasonably designing the structure of ether solvent molecules, the stability of electrolyte to Gr anode and S cathode can be simultaneously achieved, which is expected to be applied to LISBs with long cycle life.

Herein, we propose a molecule structure design strategy, by increasing steric hindrance and electronegativity of ether solvent molecules, to weaken the Li^+^‐solvent binding energy and accelerate the Li^+^ de‐solvation process, further preventing solvent co‐intercalation with Li^+^. Three different electrolytes are prepared for comparison, including 1.0 m lithium bis(trifluoromethanesulphonyl)imide (LiTFSI)‐DOL/DME (named DME‐based electrolyte), 1.0 m LiTFSI‐DOL/1, 2‐Diethoxyethane (DEE) (named DEE‐based electrolyte) and 1.0 m LiTFSI‐DOL/1, 2‐(1, 1, 2, 2‐Tetrafluoroethoxy) ethane (TFEE) (named TFEE‐based electrolyte). Theoretical calculation results and Raman spectra demonstrate that from DME, DEE to TFEE, the Li^+^‐solvent binding energy is gradually reduced along with the increment in steric hindrance and electronegativity. Furthermore, the weak solvation power in TFEE‐based electrolyte renders more TFSI^−^ anions occupying Li^+^ solvation structure and results in generating a stable anion‐derived SEI layer formed on Gr anode surface. Therefore, good reversibility and cycling stability of Gr/Li half‐cells are obtained in TFEE‐based electrolyte, revealing a stable specific capacity of ≈340 mAh g^−1^ at 0.2 C and a high average CE of above 99% within 100 cycles. Simultaneously, due to the weak Li^+^‐TFEE binding energy that can effectively suppress the shuttle effect of LiPSs, TFEE‐based electrolyte is a very promising electrolyte for Li‐S batteries. Therefore, when applying TFEE‐based electrolyte in a LISB coin cell consisting of lithiated graphite (LG) anode (≈4.3 mg cm^−2^) and S@C cathode (≈0.8 mg cm^−2^ S) (N/P ratio ≈1.1), a stable and reversible capacity of ≈770 mAh g^−1^ and a high average CE of 93% are obtained within 200 cycles at 0.1 C. Furthermore, TFEE‐based electrolyte proceeds to a LISB pouch cell to evaluate its practical applicability, which is an unprecedented study. A LISB pouch cell of 0.15 Ah was prepared with S@C cathode (≈2.1 mg cm^−2^ S), LG anode (≈10.5 mg cm^−2^) (N/P ratio ≈1.1) and 2.0 g TFEE‐based electrolyte. It delivers an initial discharge capacity of 802.3 mAh g^−1^ at 0.025 C, and still releases a stable capacity of 499.1 mAh g^−1^ with a high CE of 95.9% after 120 cycles.

## 2 Results and Discussion

### 2.1 Design Logic of Solvent Molecular Structure

**Figure 1:**
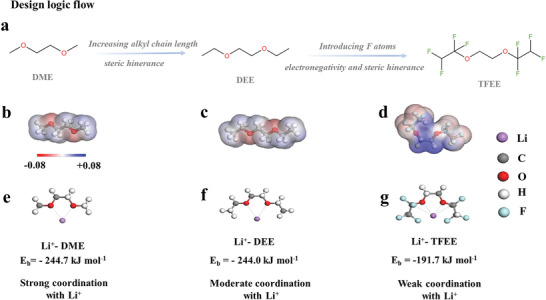
Step‐by‐step design principle. a) Logical flow, starting from DME, for the design of the molecular structure. b–d) Electrostatic potential of DME, DEE, TFEE molecule, respectively. e–g) Coordination of geometrical configuration and binding energy between Li^+^ and DME, DEE, and TFEE molecules, respectively.

It is well known that weakening the coordination ability of solvent with Li^+^ can fasten Li^+^ de‐solvation process and advance the compatibility with Gr anode.^[^ ^25^ ^]^ From this point of view, we first increase the alkyl chain length of the DME molecule to obtain a DEE molecule. Then, we furtherly substitute H atoms at the α, β position of DEE molecular bone by F atoms with large electron‐withdrawing and steric‐hindrance, to obtain a TFEE molecule (**Figure** **1**a). The physical properties of the three solvents and DOL are seen in Table S1 (Supporting Information). Electrostatic potential diagrams of DME, DEE, and TFEE molecules are shown in Figure 1b–d, respectively, displaying a strong interaction between Li^+^ and O atoms or Li^+^ and F atoms that the negative charge is more located on O and F atoms (red part in the calculated electrostatic potential diagrams). The binding energy (E_b_) of three ether molecules with Li^+^ is obtained by Density functional theory (DFT) calculations, following the order: E_b_ (Li^+^‐DME)  > E_b_ (Li^+^‐DEE) > E_b_ (Li^+^‐TFEE), corresponding to −244.7 kJ mol^−1^, −244.0 kJ mol^−1^ and −191.7 kJ mol^−1^, respectively (Figure 1e–g). As expected, the theoretical calculation results prove that TFEE solvent has the weakest coordination ability with Li^+^, whose E_b_ absolute value is much lower than that of DME and DEE solvents.

### 2.2 Physicochemical Properties and Li^+^ Solvation Structures of Electrolytes

**Figure 2:**
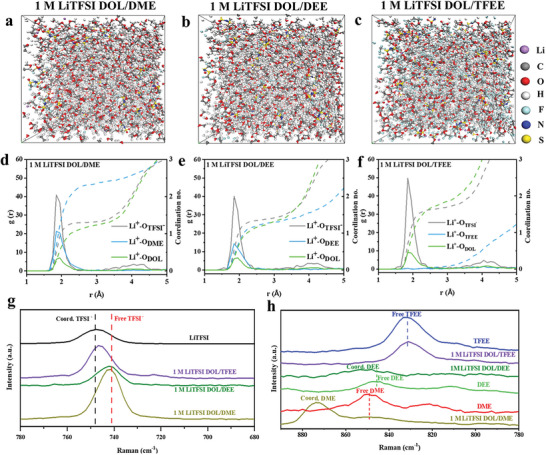
Computational and experimental analysis of Li^+^ solvation structure. Snapshot and RDF curves obtained from MD simulations of a,d) 1.0 m LiTFSI‐DOL/DME, b,e) 1.0 m LiTFSI‐DOL/DEE, c,f) 1.0 m LiTFSI‐DOL/TFEE. Raman spectra of g) different electrolytes and pure LiTFSI salt, and h) different electrolytes and solvents.

The binding energy of Li^+^ and solvents greatly affects the physicochemical properties and Li^+^ solvation structure of electrolytes. Viscosity and ionic conductivity of three electrolytes using 1.0 m LiTFSI salt and different solvents (DOL/DME, DOL/DEE, DOL/TFEE) are shown in Figure S1 (Supporting Information), demonstrating similar viscosity in three electrolytes but a decrease in ionic conductivity as the Li^+^ solvation energy decreases, which is owing to the weak dissociation ability of TFEE solvent to Li salt. Li^+^ solvation structures in three electrolytes were investigated by both computational and experimental methods. Molecular dynamics (MD) simulations were conducted to obtain Radial distribution function (RDF) curves of Li^+^ with TFSI^−^ and solvents (**Figure** **2**a–f). The simulation results reveal that the coordination number of solvents with Li^+^ follows the trend of DME > DEE > DOL > TFEE, and the coordination number of TFSI^−^ with Li^+^ is relatively enhanced along with the weakening coordination strength of solvents with Li^+^.

Raman spectra of the three electrolytes are consistent with these MD results, shown in Figure 2g,h. It is well known that upon Li salt dissolution, Raman peaks of Li salt anions undergo a significant red shift due to the weakened cation‐anion interaction and enhanced cation‐solvent interaction.^[^ ^20^, ^27^ ^]^ Figure 2g manifests that the S‐N‐S stretching vibration peak of TFSI^−^ is located at 748 cm^−1^ in pure LiTFSI salt due to strong Li^+^‐TFSI^−^ coordination. The peak is shifted to 742 cm^−1^ in DME‐based electrolyte, to 743 cm^−1^ in DEE‐based electrolyte, and to 747 cm^−1^ in TFEE‐based electrolyte.^[^ ^21^ ^]^ Fourier transform infrared (FTIR) spectra of TFSI^−^ also follows the same pattern (Figure S2, Supporting Information), ‐CF_3_ characteristic peak of TFSI^−^ is located at 1198 cm^−1^ in pure LiTFSI salt.^[^ ^28^ ^]^ The peak is red shifted to 1188 cm^−1^ in DME‐based electrolyte, to 1190 cm^−1^ in DEE‐based electrolyte, and to 1193 cm^−1^ in TFEE‐based electrolyte. These results demonstrate that Li^+^‐TFSI^−^ interaction gets stronger from DME, DEE to TFEE due to their gradual weakening solvating power with Li^+^. As shown in Figure 2h, the typical C─O stretching vibration peak of pure DME solvent locates at 848.3 cm^−1^, but the peak obviously blue shifts to 873.5 cm^−1^ arising from coordination of DME molecules with Li^+^, ^[^ ^27^ ^]^ indicating that DME molecules have a strong solvating Li^+^ ability. The characteristic vibration peak of pure DEE solvent locates at 846.5 cm^−1^, which blue shifts a little to 850.2 cm^−1^ in DEE‐based electrolyte, indicating a reducing Li^+^‐DEE coordination interaction. The characteristic vibration peak of pure TFEE solvent locates at 830 cm^−1^, which scarcely shifts in TFEE‐based electrolyte, implying the very weak solvating power of TFEE with Li^+^. All the above results confirm that by tuning molecule structure from DME, DEE to TFEE, more TFSI^−^ anions, and less solvent molecules participate in the Li^+^ solvation structure.

### 2.3 Compatibility with Graphite Electrode

The Li^+^ solvation structure of electrolytes influences Li^+^ transfer ability, which can be evaluated by Li^+^ transference number. As shown in Figure S3 (Supporting Information), the values of Li^+^ transference number obtained in three electrolytes are 0.32 for DME‐based electrolyte, 0.43 for DEE‐based electrolyte, and 0.62 for TFEE‐based electrolyte, respectively. This result indicates a faster Li^+^ transfer characteristic is obtained in TFEE‐based electrolyte.

**Figure 3:**
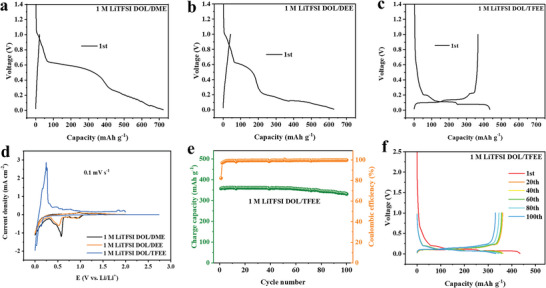
Evaluation of compatibility of electrolytes with Gr electrode and cycling performance in Gr/Li half‐cells with 1.0 m LiTFSI‐DOL/TFEE electrolyte. a–c) The initial discharge‐charge profiles at 0.1 C in 1.0 m LiTFSI‐DOL/DME, 1.0 m LiTFSI‐DOL/DEE, and 1.0 m LiTFSI‐DOL/TFEE electrolyte, respectively. d) CV profiles of Gr/Li half‐cells in different electrolytes between 0 and 2 V at a scanning rate of 0.1 mV s^−1^. e) Cycling performance and f) discharge‐charge profiles of Gr/Li half‐cell in 1.0 m LiTFSI‐DOL/TFEE electrolyte at 0.2 C, after initial 3‐cycles activation at 0.1 C.

The weakly solvating power is also beneficial for fastening Li^+^ de‐solvation process, which can suppress the co‐intercalation of Li^+^‐solvent complex, hence TFEE‐based electrolyte is expected good compatibility with Gr anode. Gr/Li half‐cells were fabricated with the three electrolytes to investigate their compatibility with Gr anode. As shown in **Figure** **3**a, the Gr/Li half‐cell with DME‐based electrolyte delivers a non‐characteristic discharge voltage plateau during 1.0–0.2 V with ≈720 mAh g^−1^ of initial discharge capacity and nearly zero of charge capacity. That means severe co‐intercalation of Li‐DME complex occurring during the initial discharge process, leading to irreversible structure collapse of Gr, which is consistent with previous reports.^[^ ^29^ ^]^ Similarly, the Gr/Li half‐cell with DEE‐based electrolyte also shows a non‐characteristic discharge voltage plateau during 1.0–0.2 V with a high initial discharge capacity of ≈630 mAh g^−1^ followed by a much low initial charge capacity of ≈50 mAh g^−1^ (Figure 3b). As expected, the Gr/Li half‐cell with TFEE‐based electrolyte delivers a typical discharge/charge profile of Gr with an initial discharge capacity of 434.9 mAh g^−1^ and an initial charge capacity of 357.9 mAh g^−1^ (Figure 3c), demonstrating good compatibility with Gr and high electrochemical reversibility. Cyclic voltammetry (CV) curves of the Gr/Li half‐cells using the three electrolytes illustrate the same results (Figure 3d). The TFEE‐based electrolyte exhibits reversible lithiation/delithiation peaks, but the DME and DEE‐based electrolytes display apparent co‐intercalation peaks between 1.0 and 0.2 V in. In addition, the cycling performance of the Gr/Li half‐cells using TFEE‐based electrolyte was tested, shown in Figure 3e,f, revealing good cycling stability with a stable and high specific capacity of ≈340 mAh g^−1^ at 0.2 C (1 C = 340 mA g^−1^) and a high average CE of above 99%.

**Figure 4:**
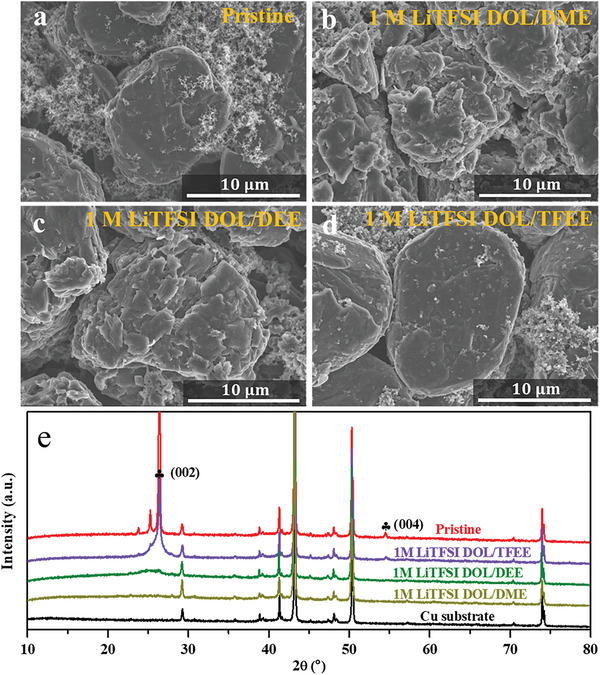
a–d) SEM images of Gr electrodes: a) pristine and cycled in b) 1.0 m LiTFSI‐DOL/DME, c) 1.0 m LiTFSI‐DOL/DEE, d) 1.0 m LiTFSI‐DOL/TFEE. e) XRD spectra of pristine and cycled Gr electrodes. (002) and (004) are corresponding to planes of Gr. All the cycled Gr electrodes were collected at delithiated states from Gr/Li half‐cells.

In order to investigate the structure of Gr electrodes cycled in different electrolytes, SEM (Scanning electron microscope) and XRD (X‐ray diffraction) are used to characterize Gr electrode. For pristine Gr electrode, a smooth and complete granular morphology is observed (**Figure** **4**a) and a typical XRD peak appear at 26.4° corresponding to the (002) planes of Gr (Figure 4e). For the Gr electrode cycled in DME and DEE‐based electrolytes, a large number of cracks appear on the surface of Gr electrode (Figure 4b,c), and the typical XRD peak at 26.4° disappears (Figure 4e), indicating the Gr structure is severely destroyed by the co‐intercalation of Li^+^‐DME or Li^+^‐DEE complexes. For the Gr electrode cycled in TFEE‐based electrolyte, the surface morphology is same as the pristine one (Figure 4d), and the sharp XRD peak at 26.4° still remains (Figure 4e), implying the Gr structure is well‐maintained due to the weakly solvating power of TFEE solvent fastens de‐solvation process of Li^+^ and inhibits the co‐intercalation phenomenon of Li^+^‐solvent complex.

### 2.4 SEI Chemistry on Gr Electrode Surface

**Figure 5:**
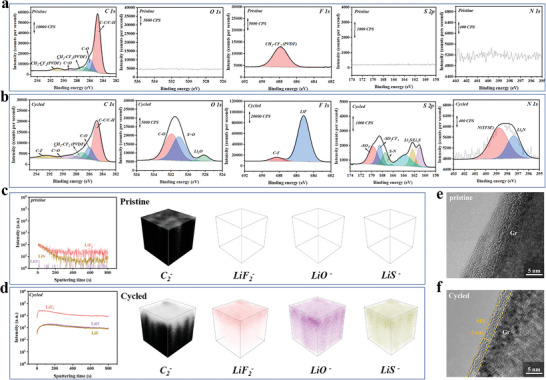
Characterization of pristine and cycled Gr surface. XPS spectra of C 1s, O 1s, F 1s, S 2p, N 1s on the surface of a) pristine and b) cycled Gr electrode in 1.0 m LiTFSI‐DOL/TFEE electrolyte. TOF‐SIMS depth profiles and distributions of C_2_^−^, LiF_2_^−^, LiO^−^, LiS^−^ species on the surface of c) pristine and d) cycled Gr electrode in 1.0 m LiTFSI‐DOL/TFEE electrolyte. HRTEM images of e) pristine and f) cycled Gr in 1.0 m LiTFSI‐DOL/TFEE electrolyte.

The more anions participating Li^+^ solvation structure in TFEE‐based electrolyte contributes to a stable anion‐derived SEI layer, which is responsible for good cycling stability of Gr/Li half‐cell. To analyze the SEI layer on Gr electrode, the Gr/Li half‐cell was disassembled after 20 cycles in TFEE‐based electrolyte at 0.2 C and the chemical species of the SEI layer on Gr electrode surface were characterized by X‐ray photoelectron spectroscopy (XPS). Compared to the pristine Gr electrode (**Figure** **5**a), Figure 5b shows the SEI layer on cycled Gr electrode surface is mainly composed of ‐CF_3_ (292.6 eV), Li_2_O (528.5 eV), LiF (685.1 eV), sulfides (Li_2_NSO_2_CF_3_/LiSO_2_CF_3_ (169.8‐167.4 eV), Li_2_S/Li_x_S (163.8–161.1 eV)), and Li_3_N (397 eV),^[^ ^21^ ^]^ demonstrating the anions are priorly reduced to form a stable SEI mainly composed of abundant inorganic components on Gr surface, which can help to stabilize the electrode/electrolyte interface and avoid further interfacial side reactions.^[^ ^23^ ^]^ The result is also supported by Time‐of‐flight secondary ion mass spectrometry (TOF‐SIMS). As shown in Figure 5c,d, LiF_2_^−^, LiO^−^, and LiS^−^ secondary ions can be observed on the surface and inner structure of cycled Gr electrode in TFEE‐based electrolyte, also demonstrating the SEI layer is mainly composed of TFSI^−^ anion decomposition products.

Besides, a high‐resolution transmission electron microscope (HRTEM) was used to further investigate the thickness of SEI layer on Gr electrodes cycled in TFEE‐based electrolyte. As shown in Figure 5e,f, an obviously layered structure and clean surface are seen in the pristine Gr (Figure 5e), and a homogeneous and thin SEI layer (≈3.0 nm) is observed on the surface of the cycled Gr (Figure 5f), also demonstrating a superior SEI layer formed in TFEE‐based electrolyte. In a word, the weakened Li^+^/TFEE coordination promotes more TFSI^−^ anions to participate in Li^+^ solvation structure, inducing a robust and anion‐derived SEI layer generated on Gr electrode surface, which contributes to improve the cycle stability of Gr electrode.

### 2.5 Electrochemical Performance of LISB Coin Cells and LISB Pouch Cells

It is well known that there exists a shuttle effect in Li‐S batteries which is arising from the intermedia long‐chain LiPSs dissolving in the electrolyte, diffusing to the Li anode and reacting with Li metal anode to generate short‐chain LiPSs. The short‐chain LiPSs either deposit on Li metal anode or diffuse back to the cathode to be oxidized to generate long‐chain LiPSs. The process continuously take place between S cathode and Li metal anode, which leads to low CE and fast capacity loss due to the generation of dead S. If the solubility of LiPSs is reduced in the electrolyte, the diffusion of LiPSs and the reaction with Li metal anode will be inhibited, thus the shuttle effect will be suppressed.

To evaluate the shuttle situation of LiPSs, stoichiometric amounts of Li_2_S and sulfur were added to the three electrolytes to generate 2 m Li_2_S_8_. As shown in Figure S4 (Supporting Information), after 48 h of stirring, DME‐based electrolyte exhibits a dark‐brown color, DEE‐based electrolyte exhibits a reddish‐brown color, and TFEE‐based electrolyte only turns into orange with lots of insoluble substances. That demonstrates LiPSs species are highly dissolving in DME‐based electrolyte, the solubility of LiPSs is a little suppressed in DEE‐based electrolyte, and the solubility is significantly suppressed in TFEE‐based electrolyte because of the weak Li^+^‐TFEE binding energy. Hence TFEE‐based electrolyte is expected to have better suppression ability to the shuttle effect of LiPSs than DME‐based electrolyte and DEE‐based electrolyte.

To evaluate whether the TFEE‐based electrolyte can suppress the shuttle effect of LiPSs, S@C/Li half‐cells with the three electrolytes were first assembled and the cycle performance was tested. Figure S5 (Supporting Information) shows that S@C/Li half‐cell with TFEE‐based electrolyte exhibits an initial discharge capacity of 1120.5 mAh g^−1^ and maintains 716.8 mAh g^−1^ after 100 cycles at 0.1 C with the average CE of ≈95%, the capacity retention rate is ≈64%. However, the S@C/Li half‐cell with DEE‐based electrolyte shows a low average CE of ≈70% and low capacity retention rate of ≈20% after 100 cycles, and the S@C/Li half‐cell with DME‐based electrolyte displays the worst CE and capacity retention rate. The enhanced cycle performance and CE of S@C/Li half‐cell with TFEE‐based electrolyte is  ascirbed to the weakly solvating ability of TFEE inhibiting the dissolution of LiPSs in the electrolyte, hence the accompanying shuttle effect is greatly suppressed.

In addition, the surface morphologies of cycled Li metal anodes in three electrolytes are also measured to investigate the shuttle situation of LiPSs. As shown in Figure S6 (Supporting Information), Li metal cycled in DME‐based electrolyte exhibits a more rough and pockmarked surface, while the surface of Li metal cycled in DEE‐based electrolyte is more even with part pockmarks. In contrast, the surface of Li metal cycled in TFEE‐based electrolyte is the  smoothest without obvious pockmarks. This illustrates that serious lithium corrosion occurs in DME‐based electrolyte due to the intense shuttle effect of LiPSs, but lithium corrosion hardly occurs in TFEE‐based electrolyte, further demonstrating the shuttle effect of LiPSs is efficiently restrained in TFEE‐based electrolyte.

**Figure 6:**
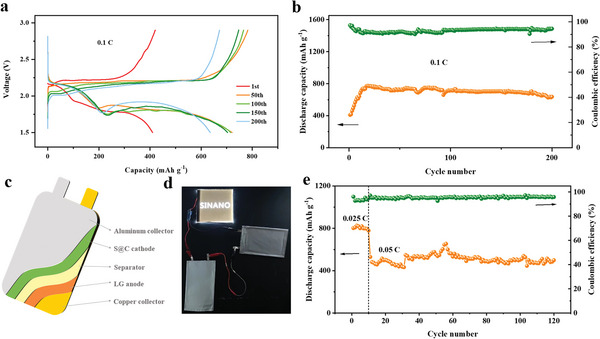
Cycling performance evaluation of LISB coin cells and LISB pouch cells in 1.0 m LiTFSI‐DOL/TFEE electrolyte (N/P capacity ratio ≈1.1). a) Discharge‐charge profiles at different cycles and b) cycling performance of LISB coin cells in 1.0 m LiTFSI‐DOL/TFEE electrolyte within 200 cycles. c) Schematic diagram of the structure of LISB pouch cell. d) A digital photo of the LED light that is lit by LISB pouch cells. e) Cycling performance of LISB pouch cells in 1.0 m LiTFSI‐DOL/TFEE electrolyte within 120 cycles after activation at room temperature.

The above results demonstrate that TFEE‐based electrolyte exhibits good compatibility with Gr anode and also greatly inhibits shuttle effect of LiPSs, hence the electrolyte is expected to be very suitable for Gr‐based LISBs. To mate the capacity of the S@C cathode and LG anode well, a LG/Li cell was assembled after pre‐lithiation process and the specific capacity of LG electrode was tested by directly charged to 1.0 V at 0.1 C. As shown in Figure S7 (Supporting Information), the LG electrode exhibits a charge specific capacity of 349.7 mAh g^−1^. Based on the specific capacity data of S@C cathode and LG anode, a LISB coin cell is constructed using a LG anode coupled with a S@C cathode under a N/P ratio ≈1.1. **Figure** **6**a depicts the discharge‐charge curves of the LISB coin cell in the 1st, 50th, 100th,150th, and 200th cycles. The discharge‐charge profiles at 0.1 C (1 C = 1675 mA g^−1^) show typically two discharge plateaus at voltage of 2.2 and 1.7 V, respectively, which are ascribed to “extract” Li^+^ from graphite.^[^ ^29^ ^]^ The LISB coin cell delivers excellent cycle stability within 200 cycles with a stable and reversible capacity of ≈770 mAh g^−1^ and high average CE of 93% (Figure 6b).

Furthermore, we proceeded to assemble a LISB pouch cell (0.15 Ah) with 2.0 g TFEE‐based electrolyte to evaluate the practical application potential under a strict condition (S loading of ≈2.1 mg cm^−2^, Gr loading of ≈10.5 mg cm^−2^, N/P ratio ≈1.1). The structure of this pouch cell is shown in Figure 6c and the other design parameters are shown in Table S2 (Supporting Information). As shown in Figure 6d, the LED light can be successfully lit by two LISB pouch cells in series. Meanwhile, this LISB pouch cell also delivers an initial discharge capacity of 802.3 mAh g^−1^ at 0.025 C, and still releases a stable capacity of 499.1 mAh g^−1^ with a high CE of 95.9% after 120 cycles (Figure 6e). The rate performance of the LISB pouch cell is also investigated in Figure S8 (Supporting Information), the discharge capacity is 825.4 mAh g^−1^ at 0.025 C, 616 mAh g^−1^ at 0.05 C, 444.1 mAh g^−1^ at 0.15 C, 310.1 mAh g^−1^ at 0.3 C, 215.3 mAh g^−1^ at 0.4 C and 143.2 mAh g^−1^ at 0.5 C. Apparently, a higher polarization is seen in the discharge plateaus along with the rate increasing.

It is noteworthy that comparing with recently reported works in Gr anode‐based LISBs (Table S3, Supporting Information), this work presents a higher capacity retention of 82.2% among 200 cycles in LISB coin cell, and exhibits an unprecedented LISB pouch cell configuration under strict conditions (S loading of ≈2.1 mg cm^−2^, Gr loading of ≈10.5 mg cm^−2^, N/P ratio ≈1.1) with good cycling stability and long lifespan. This is owing to TFEE‐based electrolyte can effectively suppress the shuttle effect of LiPSs, being compatible to the Gr anode well, and can form a stable SEI layer on Gr anode surface. This work may be a significantly important breakthrough for future application of long‐life LISBs.

## 3 Conclusions

In summary, this work provides a solvent molecular structure design strategy to weaken the coordination of solvents with Li^+^ by steric hindrance and electronegativity, which effectively improves Li^+^ de‐solvation kinetics, suppresses co‐intercalation of ether solvents into Gr electrode, and inhibits shuttle effect of LiPSs. The resulting 1.0 m LiTFSI‐DOL/TFEE electrolyte exhibits a unique Li^+^ solvation structure with more TFSI^−^ anions, inducing the generation of a robust and anion‐derived SEI layer on Gr electrode surface. This SEI layer is mainly composed of inorganic components, which effectively stabilizes the Gr anode and avoid further side reactions between anode and electrolyte. Therefore, Gr anode‐based LISBs (N/P ≈1.1) with 1.0 m LiTFSI‐DOL/TFEE electrolyte exhibits good cycling performance. After 200 cycles, the LISB coin cell shows a stable and reversible capacity of ≈770 mAh g^−1^ and a high average CE of 93%. Furthermore, A LISB pouch cell with a high Gr loading of ≈10.5 mg cm^−2^ is first reported to deliver an initial discharge capacity of 802.3 mAh g^−1^ at 0.025 C, and still releases a stable capacity of 499.1 mAh g^−1^ with a high CE of 95.9% after 120 cycles. This work provides a promising guidance for achieving long‐life LISBs in the future from the electrolyte solvent molecule structural tune.

## 4 Experimental Section

### Electrode Fabrication

The sulfur/carbon nanotube (S/CNT) composite was prepared by infiltrating the sublimed S (98%, Aladdin) into the CNT (battery level, Shandong Dazhan Nanomaterial Co., Ltd.) via a simple melt‐diffusion method. CNT and pure S were mixed with a mass ratio of 1:3 by ball milling for 30 min. Subsequently, the mixture was transferred into a sealed vessel and heated at 155 °C for 4 h, followed by heating for another 2 h at 300 °C. After cooling down to room temperature, the S/CNT composite was obtained.

The 19 wt.% DMAC aqueous solution was prepared by mixing solvent N, N‐Dimethylacetamide (DMAC, battery level, Adamas), and deionized water. The S@C cathode was prepared as follows: the S/CNT composite active material, conductive agent (including three materials, Super‐P (battery level, Termeco GMBH): CNT: graphene (battery level, Knano Graphene Technology Co., Ltd.) was corresponding to a mass ratio of 6:2:2 in the conductive agent system), and LA133 binder (battery level, Shanghai Titan Scientific Co., Ltd.) were mixed at 8:1:1 (by mass) in above DMAC solution to obtain a homogeneous slurry with a solid content of 25 wt.%. The slurry was spread on carbon‐coated aluminum foil and dried at 80 °C for 6 h. S@C electrodes were prepared with ≈0.8 mg cm^−2^ S for coin cells and ≈2.1 mg cm^−2^ S for pouch cells.

Gr electrodes were prepared by mixing 90 wt.% of Gr (battery level, China Yuexing Co., Ltd.) as active material, 5 wt.% of acetylene black (AB, battery level, Shenzhen Kejing STAR Technology Company) as a conductive agent, and 5 wt.% Poly(vinylidene fluoride) (PVDF, battery level, Solvay (Shanghai) Co., Ltd.) as binder in nonaqueous solvent N‐methyl pyrrolidone (NMP, 98%, Aladdin), then cast onto copper foil, and dried at 80 °C for 6 h. Gr electrodes were prepared with a loading level of ≈3.5 mg cm^−2^ for coin cells. LG electrodes were prepared by making prepared Gr electrodes contact directly with Li foils with the presence of 1.0 m LiTFSI‐DOL/TFEE electrolyte under pressure for 6 h. The loading level is ≈4.3 mg cm^−2^ for coin cells and ≈10.5 mg cm^−2^ for pouch cells. For practical conditions, the preparation of LG electrodes in pouch cells is different from laboratory coin cells. Gr electrodes attached with an equal stoichiometric ratio of lithium strips were assembled into the pouch cells as they would normally be. The LG electrodes were obtained after the placement of pouch cells in the oven for 48 h under pressure at 40 °C.

### Preparation of the Electrolytes

The homogeneous and transparent electrolytes were made by dissolving 1.0 m LiTFSI (98%, Jiangsu Guotai Chaowei New Material) in mixtures of DOL (98%+, Alfa Aesar)/DME (99%, Sigma‐Aldrich) (volume ratio of 1:1), or in mixtures of DOL/DEE (99%+, Adamas) (volume ratio of 1:1), or in mixtures of DOL/TFEE (99%, Apollo Scientific) (volume ratio of 1:1). Lithium sulfide (Li_2_S, 99.5%, Aladdin) and sublimed S were put into electrolytes for the investigation of Li_2_S_x_ species’ dissolution. All the electrolytes were prepared in a glove‐box with argon atmosphere (H_2_O < 1 ppm) at room temperature.

### Characterizations

The ionic conductivities of electrolytes were measured by METTLER TOLEDO S400 SevenExcellence™ measuring instrument using Inlab 741‐ISM conductive electrode. The viscosities of prepared electrolytes were evaluated by Haake RS6000 Rotational Rheometer with P25 CS L rotor at 25 °C. Raman spectroscopy (LABRAM, HR) with a laser of 532 nm and FTIR spectroscopy (Thermo Scientific Nicolet 6700 spectrometer) were used to investigate the coordination information of ions and molecules in different electrolytes. The lattice structures of graphite were examined by XRD (Bruker D8 diffractometer) with a 2θ scale of 10–80° and at a scanning rate of 2° min^−1^.

The morphologies of the Gr electrode surface were measured using a SEM (S4800, Hitachi, Japan) with a working voltage of 5 KV and HRTEM (Tecnai G2 F20 S‐TWIN). Samples for SEM analysis were first rinsed in dimethyl carbonate (DMC) and dried in the glovebox. Appropriate amount of Gr electrode HRTEM samples was evenly dispersed in DMC solvent and then dripped and dried on copper net for characterization.

The SEI chemistry of graphite electrode surface was measured by XPS (VG, Altrincham, UK) on an ESCALAB 250Xi spectrometer equipped with Al Kα‐radiation and TOF‐SIMS (TOF. SIMS5‐100) in the negative mode. The XPS samples were washed by DMC, dried in an argon glovebox, and transferred into the XPS chamber by a vacuum transfer device. Sputter the Gr electrode surface and generate the secondary ions with a 0.5 keV Cs^+^ ion beam. Ingredient depth profiling analysis was carried out in the high current mode with a pulsed 30 keV Bi^3+^ ion beam. The sputtering area and analysis area was 200 × 200 µm^2^ and 50 × 50 µm^2^, respectively.

### Electrochemical Measurements

CV of Gr/Li half‐cells was measured by a Bio‐logic VMP300 Analyzer (France), and Li foil was used as the reference and counter electrodes. The Li^+^ transference number ($t_{{\mathrm{Li}}^{+}}$) was calculated using the Bruce‐Vincent method^[^ ^30^ ^]^:

(1) $$t_{Li^{+}}=\frac{I_{s}\left(\Delta V-I_{0}R_{0}\right)}{I_{0}\left(\Delta V-I_{s}R_{s}\right)}$$

where Δ*V* is the applied constant potential (10 mV) during the polarization process, *I*_0_ and *I*_s_ are the initial and steady‐state currents, and *R*_0_ and *R*_s_ are the initial and steady‐state resistance values, respectively. Electrochemical impedance spectroscopy (EIS) was in a frequency range from 7 MHz to 100 mHz. The tests were performed using Li/Li symmetric cells at room temperature.

Electrochemical tests of Gr/Li half‐cells, LISB coin cells, and LISB pouch cells were performed by a Neware battery test system (CT‐4000, Shenzhen Neware Technology Co., Ltd.). All coin cells (CR2025) were assembled by using polypropylene (PP) membrane (Celgard 2400) as the separator in an Ar‐filled glovebox with a controlled H_2_O and O_2_ level below 1 ppm.

### Theoretical Simulations

MD simulations were performed using Forcite package in Materials Studio software.^[^ ^31^, ^32^ ^]^ The amorphous cells with 46.5 × 46.5 × 46.5 Å^3^, 47.2 × 47.2 × 47.2 Å^3^, and 47.9 × 47.9 × 47.9 Å^3^ linear dimensions were adopted for simulations, corresponding to the three electrolytes with salt‐DOL‐solvent molar ratios of 60:429:289 for 1.0 m LiTFSI‐DOL/DME, 66:472:235 for 1.0 m LiTFSI‐DOL/DEE, and 69:494:195 for 1.0 m LiTFSI‐DOL/TFEE, respectively. A time step of 1.0 fs (femtosecond) was chosen. After 5 ps (picosecond) equilibration steps, statistical averages were computed from trajectories of at least 20 ps in length. Production runs were performed in an NVT ensemble and COMPASSIII force field, and the temperature was controlled using a Nosé thermostat with a target temperature of 298 K.^[^ ^33^, ^34^ ^]^

DFT calculations were performed by DMol3 module in Materials Studio software.^[^ ^35^, ^36^ ^]^ The electrostatic potential and geometry optimizations were performed with generalized‐gradient approximation/Perdew‐Burke‐Ernzerhof (GGA/PBE) functional and double numeric polarization (DNP) basis set.^[^ ^21^, ^26^ ^]^ The convergence tolerance was set to be 1.0 × 10^−5^ Ha, 2.0 × 10^−3^ Ha Å^−1^, and 5.0 × 10^−3^ Å for energy, maximum force, and maximum displacement, respectively. Binding energies of anion or solvent and Li^+^ were calculated by the following formula: E_b_ = E_total_  − E_1_ − E_2_. E_total_, E_n_ was the energy of the Li^+^‐solvent complex, single solvent, respectively.

## Conflict of Interest

The authors declare no conflict of interest.

## Supporting information

Supporting Information

## Data Availability

Research data are not shared.

## References

[1] P. G. Bruce , S. A. Freunberger , L. J. Hardwick , J. M. Tarascon , Nat. Mater. 2011, 11, 19. 22169914 10.1038/nmat3191

[2] A. Manthiram , Y. Fu , S. H. Chung , C. Zu , Y. S. Su , Chem. Rev. 2014, 114, 11751. 25026475 10.1021/cr500062v

[3] W. Xu , J. Wang , F. Ding , X. Chen , E. Nasybulin , Y. Zhang , J. G. Zhang , Energy Environ. Sci. 2014, 7, 513.

[4] X. B. Cheng , T. Z. Hou , R. Zhang , H. J. Peng , C. Z. Zhao , J. Q. Huang , Q. Zhang , Adv. Mater. 2016, 28, 2888. 26900679 10.1002/adma.201506124

[5] C. Zhang , R. Lyu , W. Lv , H. Li , W. Jiang , J. Li , S. C. Gu , G. M. Zhou , Z. J. Huang , Y. B. Zhang , J. Q. Wu , Q. H. Yang , F. Y. Kang , Adv. Mater. 2019, 31, 1904991. 10.1002/adma.201904991 31549760

[6] Y. M. Zhao , G. X. Li , Y. Gao , D. W. Wang , Q. Q. Huang , D. H. Wang , ACS Energy Lett. 2019, 4, 1271.

[7] Z. P. Jiang , L. Jin , Z. L. Han , W. Hu , Z. Q. Zeng , Y. L. Sun , J. Xie , Angew. Chem., Int. Ed. 2019, 58, 11374. 10.1002/anie.201905712 31111996

[8] J. Bae , Y. M. Qian , Yumin , Y. T. Li , X. Y. Zhou , J. B. Goodenough , G. H. Yu , Energy Environ. Sci. 2019, 12, 3319.

[9] W. Guo , W. Y. Zhang , Y. B. Si , D. H. Wang , Y. Z. Fu , A. Manthiram , Nat. Commun. 2021, 12, 3031. 34050171 10.1038/s41467-021-23155-3 PMC8163853

[10] K. X. Zhao , Q. Jin , L. Y. Zhang , L. Li , L. L. Wu , X. T. Zhang , Electrochim. Acta 2021, 393, 138981.

[11] M. Agostini , J. Hassoun , J. Liu , M. Jeong , H. Nara , T. Momma , T. Osaka , Y. K. Sun , B. Scrosati , ACS Appl. Mater. Interfaces 2014, 6, 10924. 24559093 10.1021/am4057166

[12] Y. Yang , M. T. Mcdowell , A. Jackson , J. J. Cha , S. S. Hong , Y. Cui , Nano Lett. 2010, 10, 1486. 20184382 10.1021/nl100504q

[13] J. Hassoun , B. Scrosati , Angew. Chem., Int. Ed. 2010, 49, 2371. 10.1002/anie.200907324 20191654

[14] W. D. Zhou , X. C. Xiao , M. Cai , L. Yang , Nano Lett. 2014, 14, 5250. 25158077 10.1021/nl502238b

[15] C. M. Park , J. H. Kim , H. Kim , H. J. Sohn , Chem. Soc. Rev. 2010, 39, 3115. 20593097 10.1039/b919877f

[16] J. Brückner , S. Thieme , F. Böttger‐Hiller , I. Bauer , H. T. Grossmann , P. Strubel , H. Althues , S. Spange , S. Kaskel , Adv. Funct. Mater. 2014, 24, 1284.

[17] Z. Li , S. G. Zhang , S. Terada , X. F. Ma , K. Ikeda , Y. Kamei , C. Zhang , K. Dokko , M. Watanabe , ACS Appl. Mater. Interfaces 2016, 8, 16053. 27282172 10.1021/acsami.6b03736

[18] J. Gao , M. A. Lowe , Y. Kiya Y , H. D. Abruña , J. Phys. Chem. C 2011, 115, 25132.

[19] D. P. Lv , P. F. Yan , Y. Y. Shao , Q. Y. Li , S. Ferrara , H. L. Pan , G. L. Graff , B. Polzin , C. M. Wamg , J. G. Zhang , J. Liu , J. Xiao , Chem. Commun. 2015, 51, 13454. 10.1039/c5cc05171a 26214797

[20] L. M. Suo , F. Zheng , Y. S. Hu , L. Q. Chen , Chinese Phys. B 2016, 25, 016101.

[21] Z. C. Wang , Y. Y. Sun , Y. Y. Mao , F. R. Zhang , L. Zheng , D. S. Fu , Y. B. Shen , J. C. Hu , H. L. Dong , J. J. Xu , X. D. Wu , Energy Storage Mater. 2020, 30, 228.

[22] L. L. Jiang , C. Yan , Y. X. Yao , W. L. Cai , J. Q. Huang , Q. Zhang , Angew. Chem., Int. Ed. 2021, 60, 3402. 10.1002/anie.202009738 33107707

[23] P. Ma , P. Mirmira , P. J. Eng , S. B. Son , I. D. Bloom , A. S. Filatov , C. V. Amanchukwu , Energy Environ. Sci. 2022, 15, 4823.

[24] T. Ma , Y. X. Ni , Q. R. Wang , W. J. Zhang , S. Jin , S. B. Zheng , X. Yang , Y. P. Hou , Z. L. Tao , J. Chen , Angew. Chem., Int. Ed. 2022, 61, e202207927. 10.1002/anie.202207927 35924827

[25] Y. X. Yao , X. Chen , C. Yan , X. Q. Zhang , W. L. Cai , J. Q. Huang , Q. Zhang , Angew. Chem., Int. Ed. 2021, 60, 4090. 10.1002/anie.202011482 32976693

[26] J. Holoubek , H. D. Liu , Z. H. Wu , Y. J. Yin , X. Xing , G. R. Cai , S. C. Yu , H. Y. Zhou , T. A. Pascal , Z. Chen , P. Liu , Nat. Energy 2021, 6, 303. 10.1038/s41560-021-00783-z PMC7954221 33717504

[27] S. R. Chen , J. M. Zheng , D. H. Mei , K. S. Han , M. H. Engelhard , W. G. Zhao , W. Xu , J. Liu , J. G. Zhang , Adv. Mater. 2018, 30, 1706102. 10.1002/adma.201706102 29575163

[28] L. J. Hardwick , J. A. Saint , I. T. Lucas , M. M. Doeff , R. Kostecki , J. Electrochem. Soc. 2008, 156, A120.

[29] S. R. Chen , Z. X. Yu , M. L. Gordin , R. Yi , J. X. Song , D. H. Wang , ACS Appl. Mater. Interfaces 2017, 9, 6959. 28157286 10.1021/acsami.6b11008

[30] J. Evans , C. A. Vincent , P. G. Bruce , Polymer 1987, 28, 2324.

[31] R. Gholizadeh , Y. J. Wang , Chem. Eng. Sci. 2018, 184, 62.

[32] Z. C. Wang , F. R. Zhang , Y. Y. Sun , L. Zheng , Y. B. Shen , D. S. Fu , W. F. Li , A. R. Pan , L. Wang , J. J. Xu , J. C. Hu , X. D. Wu , Adv. Energy Mater. 2021, 11, 2003752.

[33] J. F. Qian , W. A. Henderson , W. Xu , P. Bhattacharya , M. Engelhard , O. Borodin , J. G. Zhang , Nat. Commun. 2015, 6, 6362. 25698340 10.1038/ncomms7362 PMC4346622

[34] N. H. C. Lewis , Y. Zhang , B. Dereka , E. V. Carino , E. J. Maginn , A. Tokmakoff , J. Phys. Chem. C 2020, 124, 3470.

[35] B. Delley , J. Chem. Phys. 1990, 92, 508.

[36] Z. C. Wang , H. Y. Zhang , J. J. Xu , A. R. Pan , F. R. Zhang , L. Wang , R. Han , J. C. Hu , M. N. Liu , X. D. Wu , Adv. Funct. Mater. 2022, 32, 2112598.

