# Supplementary material for: Long‐Life Lithium‐Ion Sulfur Pouch Battery Enabled by Regulating Solvent Molecules and Using Lithiated Graphite Anode
Source: Adv Sci (Weinh). 2023 Sep 15;10(30):2302966. doi: 10.1002/advs.202302966 (PMC10602568; doi:10.1002/advs.202302966)
Supplement: Supplementary file 1 — Supporting Information [file ADVS-10-2302966-s001.pdf]

ADVANCED SCIENCE
Open Access

Supporting Information

for *Adv. Sci.*, DOI 10.1002/advs.202302966

Long-Life Lithium-Ion Sulfur Pouch Battery Enabled by Regulating Solvent Molecules and Using Lithiated Graphite Anode

Dan Huang, Zhicheng Wang, Ran Han, Shoulei Hu, Jiangyan Xue, Yumeng Wei, Haiqi Song, Yang Liu, Jingjing Xu, Jun Ge* and Xiaodong Wu**

Supporting Information

Long-life Lithium-ion Sulfur Pouch Battery Enabled by Regulating Solvent Molecules and Using Lithiated Graphite Anode

Dan Huang, Zhicheng Wang, Ran Han, Shoulei Hu, Jiangyan Xue, Yumeng Wei, Haiqi Song, Yang Liu, Jingjing Xu, Jun Ge,* and Xiaodong Wu**

D. Huang, Dr. Z. C. Wang, Y. Liu, Prof. J. J. Xu, Prof. X. D. Wu
School of Nano-Tech and Nano-Bionics
University of Science and Technology of China
Hefei 230026, China
E-mail: jjxu2011@sinano.ac.cn; xdwu2011@sinano.ac.cn

D. Huang, Dr. Z. C. Wang, R. Han, S. L. Hu, Dr. J. Y. Xue, Y. M. Wei, H. Q. Song, Y. Liu, Prof. J. J. Xu, Dr. J. Ge, Prof. X. D. Wu
i-lab
Suzhou Institute of Nano-Tech and Nano-Bionics (SINANO)
Chinese Academy of Sciences
Suzhou 215123, China
E-mail: jge2009@sinano.ac.cn

Table S1. Physical properties of four solvents.

Solvents	Molecular Structure	M_w/g mol^{-1}	m. p./°C	b. p./°C	Density/g ml^{-1}	ε	CAS Number
1,3-Dioxolane (1,3-DOL)	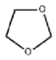	74.08	-95	75	1.06	7.34	646-06-0
1,2-Dimethoxyethane (DME)	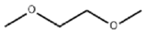	90.12	-69	85	0.867	7.2	110-71-4
1,2-Diethoxyethane (DEE)	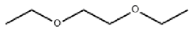	118.17	-74	121	0.842	Unknown	629-14-1
1,2-(1,1,2,2-Tetrafluoroethoxy)ethane (TFEE)	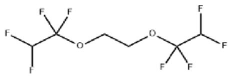	262.1	Unknown	141	1.48	Unknown	358-39-4

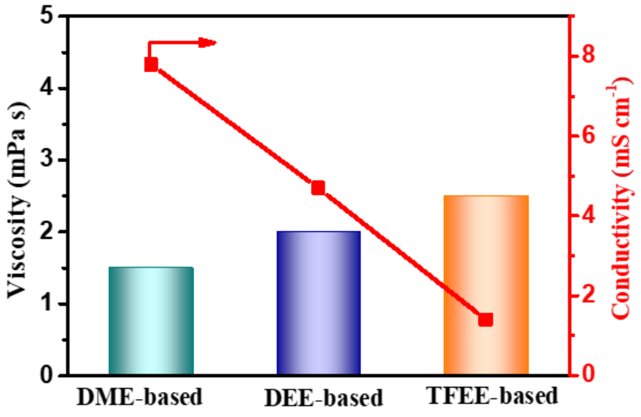

Figure S1. Viscosity and ionic conductivity of 1.0 M LiTFSI-DOL/DME, 1.0 M LiTFSI-DOL/DEE and 1.0 M LiTFSI-DOL/TFEE.

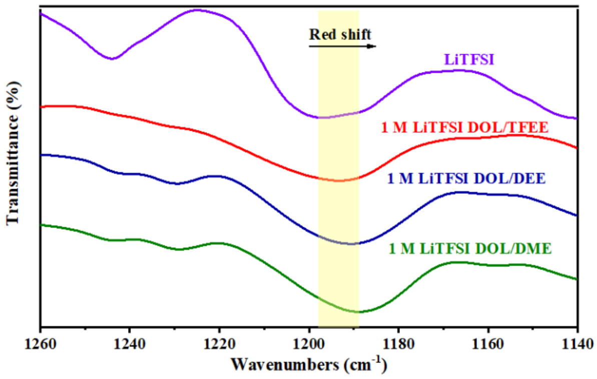

Figure S2. FTIR spectra of different electrolytes and the pure LiTFSI salt in the range of 1140-1260 cm^{-1}.

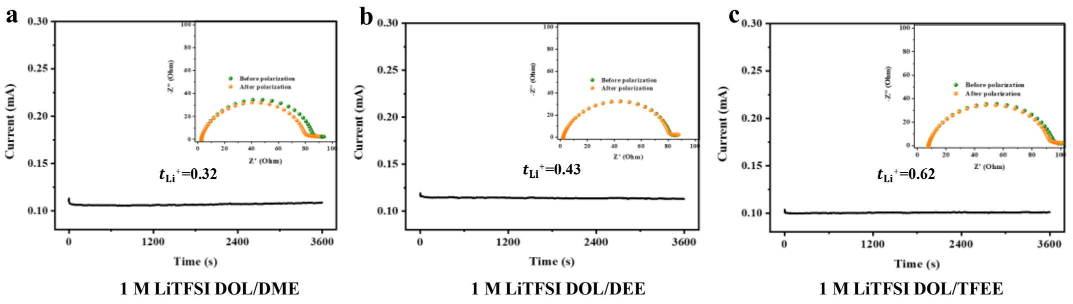

Figure S3. Li^+ transference number calculations at room temperature in (a) 1.0 M LiTFSI-DOL/DME, (b) 1.0 M LiTFSI-DOL/DEE and (c) 1.0 M LiTFSI-DOL/TFEE. The insets are Nyquist impedance plots before and after polarization used for calculations.

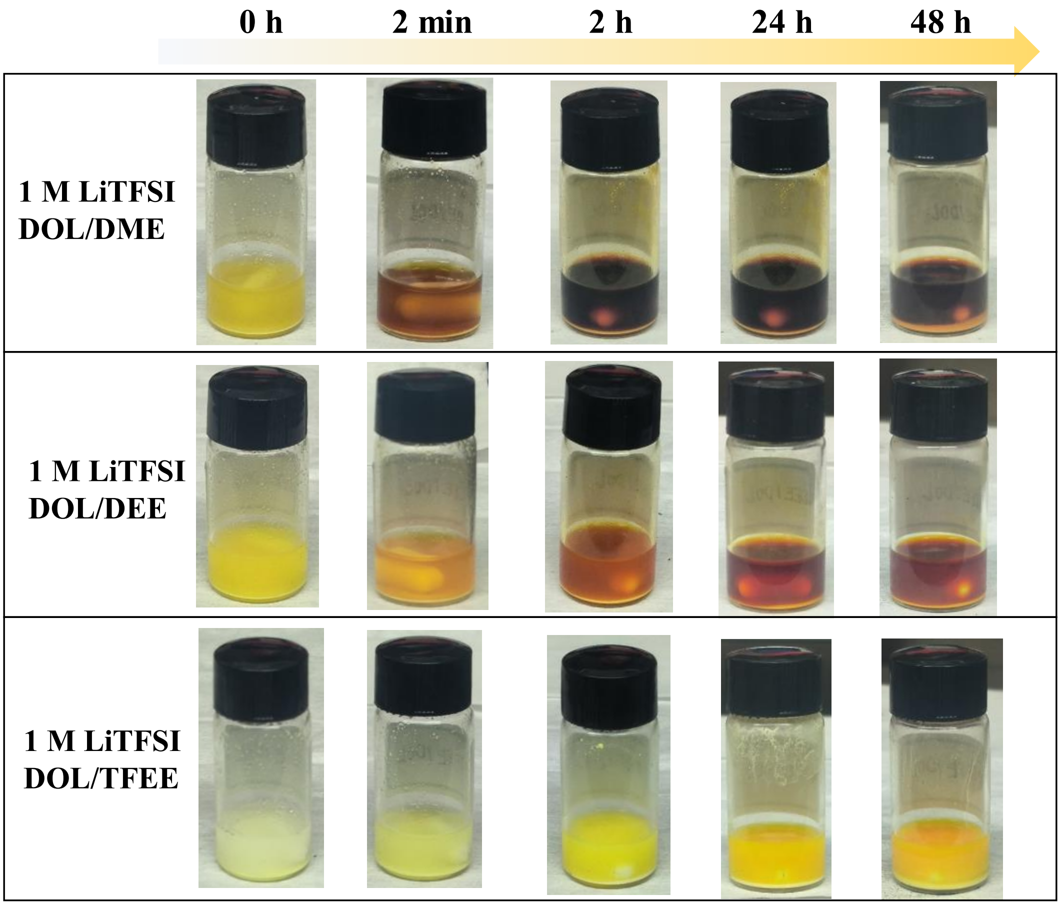

Figure S4. Photographs demonstrating the relative solubility of 2 M Li_2S_8 in three electrolytes. Stoichiometric amounts of Li_2S and sulfur are added into the three electrolytes to generate 2 M Li_2S_8 (in terms of the concentration of sulfur atoms) by stirring in the electrolytes for 0 h, 2 min, 2 h, 24 h and 48 h.

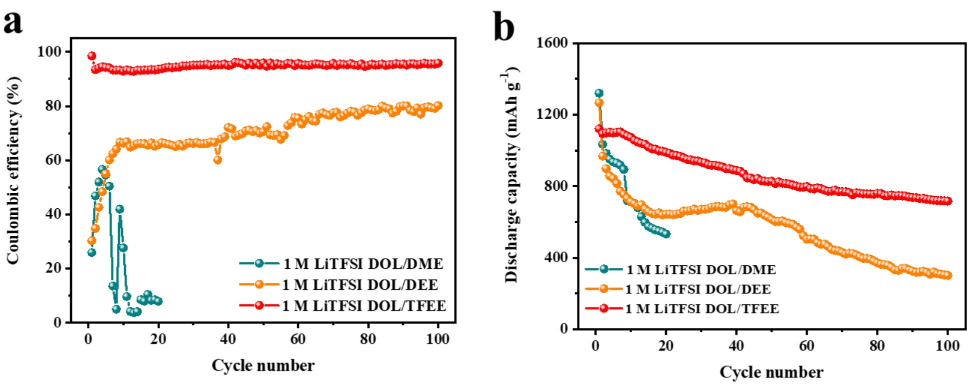

Figure S5. The Coulombic efficiency and discharge capacity of S@C/Li half-cells using different electrolyte.

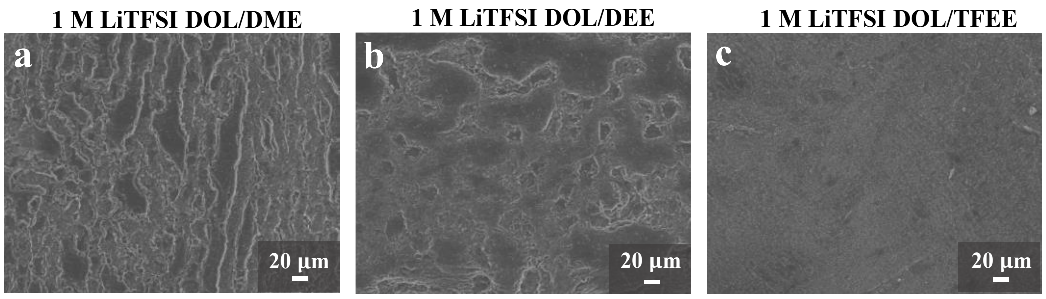

Figure S6. Surface morphologies of cycled Li metal anodes in three electrolytes from disassembled S@C/Li half-cells after 5 cycles: (a) 1.0 M LiTFSI-DOL/DME, (b) 1.0 M LiTFSI-DOL/DEE and (c) 1.0 M LiTFSI-DOL/TFEE.

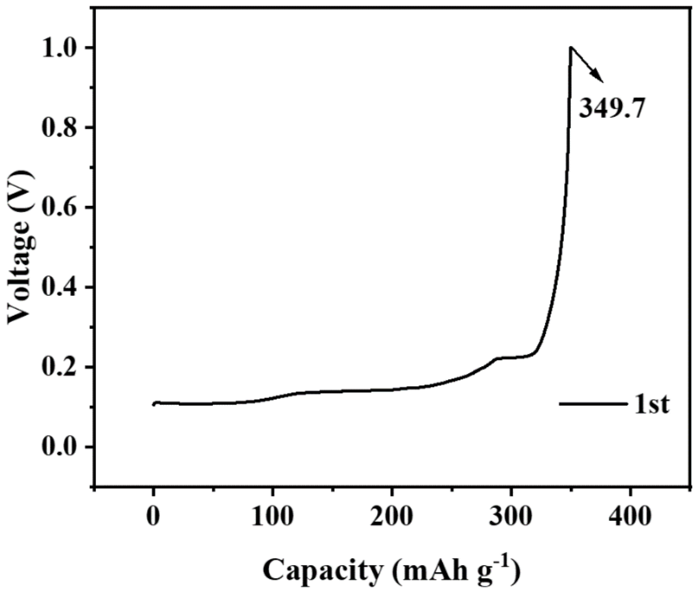

Figure S7. Initial charge profile of the LG/Li cell using TFEE-based electrolyte at 0.1 C.

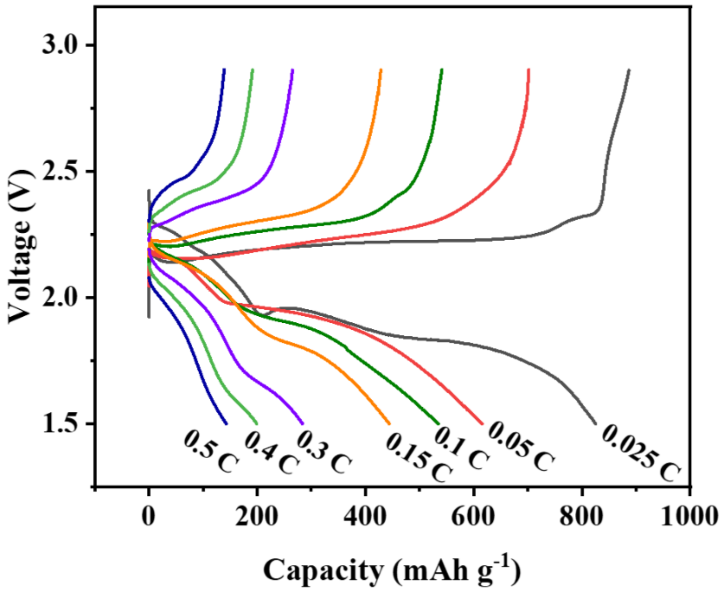

Figure S8. Rate performance of the LISB pouch cell with 1.0 M LiTFSI-DOL/TFEE electrolyte (N/P capacity ratio ≈1.1).

Table S2. Design parameters of LISB pouch cells.

Components	Design parameters		Value
S@C cathode	Active material ratio		58.5%
	Unilateral loading		≈2.1 mg cm^{-2}
	Size (length × width)		98 mm × 68 mm
LG anode	Gr anode	Active material ratio	90.0%
		Unilateral loading	10.5 mg cm^{-2}
		Size (length × width)	100 mm × 70 mm
	Li metal strip	Thickness	≈20 µm
Electrolyte	Density		1.4 g mL^{-1}
	E/S ratio		5.3 mL g^{-1}
Pouch cell	N/P ratio		≈1.1

Table S3. Comparisons of this work and recently reported systems in Gr anode-based LISBs.

Eletrolyte	Cell configuration	Active material loading (mg cm^{-2})	N/P ratio	Capacity retention	Cycles	E/S (mL g^{-1})	Pouch cell configuration	References
1 M LiTFSI DOL/TFEE (1:1, v/v)	S/LG	~0.8 in coin cell ~2.1 in pouch cell	1.1	82.2% at 1/10 C (1C=1675 mA g^{-1})	200	5.3 in pouch cells	S/LG	This work
5 M LiTFSI DOL	S/LG	2	Unknown	81.3% at 1/2 C (1C=1000 mA g^{-1})	100	Unknown	None	Chem. Commun., 2015, 51, 13454-13457
[Li(G4)$_{0.8}$][TFSA]/HFE	Li_2S/Gr	~2.2	Unknown	50.7% at 1/12 C (1C=1166 mA g^{-1})	100	Unknown	None	ACS Appl. Mater. Interfaces 2016, 8, 16053-16062
[Li(SL)$_2$][TFSA]:HFE (1:2)	Li_2S/Gr	3.65	Unknown	~69.7% at 1/8 C	300	6.2 in coin cell	None	ACS Energy Lett. 2020, 5, 1-7
1 M Li[TFSA]-G3-4.46HFE	S/LG	0.6	1.5	~64.3% at 1/20 C (1C=1672 mA g^{-1})	100	Unknown	None	J. Phys. Chem. C 2015, 119, 3957-3970
1 M LiTFSI DOL/BTFE (1:1, v/v)	S/LG	1.85	1.0-1.2	~63.1% at 1/10 C	450	Unknown	None	ACS Appl. Mater. Interfaces 2017, 9, 6959-6966
2.5 M LiTFSI+0.4 M $LiNO_3$ DOL/ DME(1/1, v/v))	S/LG	1.20	~1.73	~62.5% at 1/10 C	200	Unknown	None	ACS Energy Lett. 2018, 3, 335-340
[Li(G4)$_{0.8}$][TFSA]/HFE	Li_2S/Gr	~2.2	Unknown	50.7% at 1/12 C (1C=1166 mA g^{-1})	100	Unknown	None	ACS Appl. Mater. Interfaces 2016, 8, 16053-16062
